# Ocean acidification increases copper toxicity differentially in two key marine invertebrates with distinct acid-base responses

**DOI:** 10.1038/srep21554

**Published:** 2016-02-22

**Authors:** Ceri Lewis, Robert P. Ellis, Emily Vernon, Katie Elliot, Sam Newbatt, Rod W. Wilson

**Affiliations:** 1 College of Life and Environmental Sciences, University of Exeter, Geoffrey Pope Building, Stocker Road, Exeter, EX4 4QD, UK

## Abstract

Ocean acidification (OA) is expected to indirectly impact biota living in contaminated coastal environments by altering the bioavailability and potentially toxicity of many pH-sensitive metals. Here, we show that OA (pH 7.71; *p*CO_2_ 1480 μatm) significantly increases the toxicity responses to a global coastal contaminant (copper ~0.1 μM) in two keystone benthic species; mussels (*Mytilus edulis*) and purple sea urchins (*Paracentrotus lividus*). Mussels showed an extracellular acidosis in response to OA and copper individually which was enhanced during combined exposure. In contrast, urchins maintained extracellular fluid pH under OA by accumulating bicarbonate but exhibited a slight alkalosis in response to copper either alone or with OA. Importantly, copper-induced damage to DNA and lipids was significantly greater under OA compared to control conditions (pH 8.14; *p*CO_2_ 470 μatm) for both species. However, this increase in DNA-damage was four times lower in urchins than mussels, suggesting that internal acid-base regulation in urchins may substantially moderate the magnitude of this OA-induced copper toxicity effect. Thus, changes in metal toxicity under OA may not purely be driven by metal speciation in seawater and may be far more diverse than either single-stressor or single-species studies indicate. This has important implications for future environmental management strategies.

Ocean acidification (OA), the drop in ocean pH associated with increasing levels of carbon dioxide in the atmosphere and hence the oceans, is now widely considered to be one of the most pervasive human impacts on global marine biodiversity^1^,^2^. Recent projections published as part of the Representative Concentration Pathways (RCP) database suggest that atmospheric *p*CO_2_ levels may exceed 1000 μatm early in the next century (RCP 8.5)^3^ causing the average pH of the world’s surface waters to drop by as much as 0.43 units to around 7.73^3^,^4^. There is now a wealth of evidence that this change in ocean carbonate chemistry has the potential to impact upon the health and physiology of a wide range of marine invertebrate species^5^,^6^,^7^,^8^,^9^ with 63% of echinoderms and 51.6% of molluscs tested so far showing negative impacts of near-future OA, making them amongst the most sensitive phyla^10^.

Whilst the physiological impacts of OA for many marine biota have been widely studied, the potential for OA to interact with additional environmental stressors remains poorly understood. Changes in ocean carbonate chemistry are happening against a background of additional anthropogenically driven changes such as warming, sea level rise, increasing hypoxic and anoxic zones and chronic coastal pollution. The urgent need for ‘multi-stressor’ studies is now widely acknowledged by the OA community^11^ but to date such studies have tended to focus on combining OA with either temperature, salinity or hypoxia^12^,^13^,^14^. Of particular concern for environmental monitoring purposes is the potential for the predicted changes in ocean pH to alter the behaviour and bioavailability of historical and chronic coastal contaminants, such as metals^15^,^16^,^17^.

Metals are one of the most common types of coastal contaminant globally and are found in high concentrations in the waters and sediments of many coastal and estuarine systems^18^,^19^. For example, concentrations of total dissolved copper in U.K. coastal and estuarine waters can range from chronic low levels of 0.004 μM^20^ to as high as 1.61 μM in highly contaminated habitats^21^. OA is expected to alter the bioavailability of waterborne metals^15^ as a result of changes in their speciation in seawater, driven by the declining pH. The toxic free-ion concentration of copper (Cu^2+^) is predicted to increase by 115% in coastal waters in the next 100 years due to reduced pH^22^,^23^, while the free-ion concentration of other metals including cadmium (Cd) may decrease or be unaffected^22^,^24^,^25^,^26^. Increased metal accumulation under near-future OA conditions has been demonstrated for two bivalves species so far^27^ and for marine organisms exposed to the same nominal concentrations of any metal, greater metal toxicity effects would be predicted under near-future OA where reduced seawater pH increases free ion availability.

Whilst many transition metals including copper are essential for biological functions, elevated levels can overwhelm an organism’s antioxidant defences and induce oxidative damage of cellular components such as lipids, proteins and DNA via the production of reactive oxygen species^28^. In addition copper is known to exert a number of physiological impacts that are similar to those observed in response to exposures to OA conditions, such as growth reduction, disturbance of acid-base and osmotic regulation, and enzyme inhibition^29^,^30^. Marine animals acutely subjected to seawater with elevated *p*CO_2_ experience a corresponding extracellular acidosis^9^,^31^. Many fish and crustaceans are able to regulate these acid-base perturbations by the elevation of extracellular bicarbonate ions (HCO_3_^−^) whilst other invertebrates, such as mussels and some urchin species, are generally considered to be less able to acid-base regulate^32^,^33^. Furthermore, copper has been demonstrated to inhibit carbonic anhydrase, a vital enzyme for acid-base regulation and identified as an enzyme of interest for OA physiological studies^34^. These overlapping physiological and toxicity effects of OA and copper pollution suggest the potential for additional interactions in the responses of an organism to both stressors when exposed in combination on top of those driven purely by the metal speciation changes. Understanding these potential interactions is vital for understanding the impact of OA on coastal metal contamination and its impacts on both commercially and ecologically important biota.

We examined a suite of physiological and toxicity responses to combined OA and copper (nominal 0.1 μM) exposures in two ecologically important marine invertebrates with known sensitivities to OA as a single stressor. The common mussel *Mytilus edulis* is both an economically important shellfish species and provides a key ecosystem service, forming important substratum for many epibionts and influencing ecosystem functioning via their role in nutrient and mineral cycling^33^. The sea urchin *Paracentrotus lividus* is an ecologically important herbivore in coastal benthic habitats with additional economic importance as a food source. We used a simple factorial design to test the hypothesis that OA increases the toxicity response to copper of these two key benthic invertebrates.

## Results and Discussion

### Acid-base responses differed between mussels and urchins

**Figure 1:**
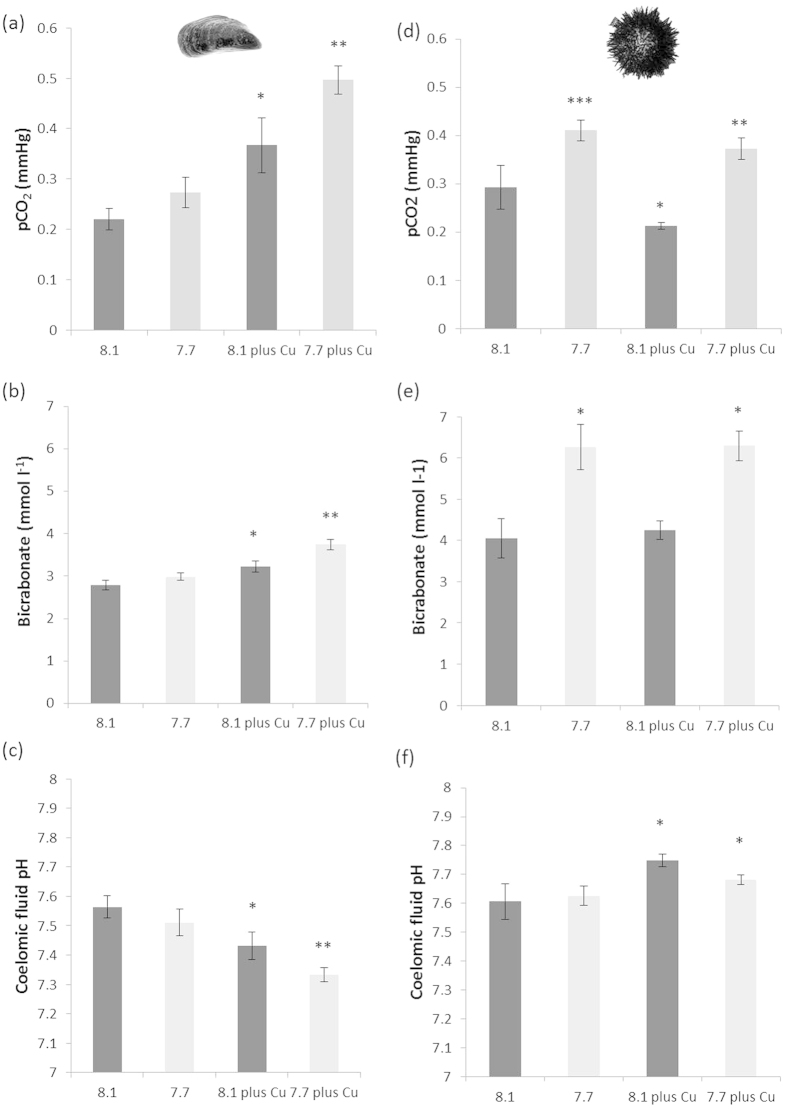
Acid-base parameters in the haemolymph of *Mytilus edulis* (**a,c,e**) and coelomic fluid of *Paracentrotus lividus* (**b,d,f**) following 14 day exposures to elevated pCO_2_ with and without the presence of nominal 0.1 μM copper; (**a,b**) haemolymph/coelomic fluid pH, (**c,d**) haemolymph/coelomic fluid bicarbonate concentrations, and (**e,f**) haemolymph/coelomic fluid pCO_2_. [N.B. *represent significant differences].

The ability to compensate for OA-induced changes in extracellular pH is believed to be a key determinant of an organisms’ ability to tolerate near-future OA^10^,^35^. Interestingly, we found very different acid-base responses between mussels and urchins to both the OA conditions and the copper exposures. In mussels, haemolymph *p*CO_2_ levels increased slightly but non-significantly with exposure to OA alone, increased further with exposure to nominal 0.1 μM copper alone and showed the greatest increase in *p*CO_2_ in the combined OA-copper exposures (Fig. 1a; two-way GLM model for OA F_1,39_ = 6.60, P = 0.014; for copper F_1,39_ = 26.73, P < 0.001; interaction term F_1,39_ = 1.32, P = 0.258). Haemolymph bicarbonate levels (HCO_3_^−^) in mussels showed a similar pattern of change in response to this elevated *p*CO_2_ (Fig. 1b), with a small but significant increase of ~0.2 mM under OA conditions and about double this increase when exposed to copper alone (Fig. 1b; two-way GLM model for OA F_1,39_ = 9.75, P = 0.004; for copper F_1,39_ = 26.57, P < 0.001; interaction term F_1,39_ = 1.95, P = 0.171). Combined exposure to OA and nominal 0.1 μM copper caused a further increase in haemolymph bicarbonate level of ~1 mM. These *p*CO_2_ changes combined with limited bicarbonate responses drove a slight but non-significant acidosis of the haemolymph in mussels exposed to OA conditions, whilst exposure to nominal 0.1 μM copper induced a stronger and significant acidosis of haemolymph from 7.56 to 7.43 (Fig. 1c). The combined OA and copper exposure caused an even greater acidosis reducing haemolymph pH to 7.33, however statistical analysis revealed this was not an interactive effect (Fig. 1c; two-way GLM model for OA F_1,39_ = 3.74, P = 0.061; for copper F_1,39_ = 15.58, P < 0.001; interaction term F_1,39_ = 0.34, P = 0.562).

The increased haemolymph *p*CO_2_ in the combined OA and copper treatment compared to the OA (no copper) treatment might be explained by changes in mussel ventilation rate as a behavioural mechanism to reduce acidosis, since they appear unable to accumulate substantial bicarbonate as a buffering mechanism. Whilst ventilation rate was not measured here, increased mussel gaping (i.e. greater amount of time ventilating the gills) under reduced seawater pH conditions has been reported elsewhere^36^. Hyperventilation in response to copper may be less likely as it could be counter-productive by increasing exposure of gills to the waterborne metal. Mussels have been shown to reduce the amount of time spent with their shells open in response to exposures to metals such as copper^37^. These results support the general consensus that mussels are not good acid-base regulators with a limited ability to buffer their haemolymph using bicarbonate^33^, instead responding to periods of hypercapnia with metabolic suppression or changes in ventilation rate.

Conversely, we found that urchins were able to employ physiological mechanisms to regulate coelomic fluid pH against the CO_2_-induced drop in external seawater pH. In sea urchins gas exchange, i.e. uptake of O_2_ and elimination of CO_2_, relies solely on a favourable diffusion gradient due to the lack of any active ventilatory mechanism, so is generally considered to be diffusion-limited^38^. Despite this inability to regulate internal *p*CO_2_ levels, urchins appear to have varying abilities to acid-base regulate depending on species^32^,^39^,^40^. In the present experiments with *P. lividus*, we observed a rise in coelomic fluid *p*CO_2_ in both the OA (alone) and OA with copper treatments, but a slight yet significant reduction in *p*CO_2_ caused by copper alone (Fig. 1d; two-way GLM model for OA F_1,32_ = 35.14, P < 0.001; for copper F_1,32_ = 6.33, P = 0.018; interaction term F_1,32_ = 0.78, P = 0.385). Coelomic fluid bicarbonate levels were significantly elevated by >2 mM in response to this elevated *p*CO_2_ in both OA treatments, whilst exposure to copper alone did not affect bicarbonate levels (Fig. 1e; two-way GLM model for OA F_1,32_ = 29.25, P < 0.001; for copper F_1,32_ = 0.07, P = 0.793; interaction term F_1,32_ = 0.06, P = 0.809).

All *P. lividus* in the present study maintained a coelomic fluid pH between 7.60 and 7.76, independent of exposure to OA conditions or elevated copper (Fig. 1f; two-way GLM model for OA F_1,32_ = 0.56, P = 0.461; for copper F_1,32_ = 9.31, P = 0.005; interaction term F_1,32_ = 1.83, P = 0.186), most likely attributed to the 48% and 55% increase, respectively, in the coelomic fluid bicarbonate levels in the two OA treatments buffering against the effect of the increased *p*CO_2_. Increased protein concentrations of coelomic fluid have also been suggested to play a role in this buffering capacity^41^ however this was not evident in our data (see below). This agrees with previous studies on *P. lividus* which have shown full compensation of coelomic fluid pH at seawater *p*CO_2_ of 1293 μatm (pH of 7.7) and a partial ability to buffer against an external *p*CO_2_ rise over a wider seawater *p*CO_2_ range (*p*CO_2_ 583–2364 μatm; pH 8.0–7.4)^32^,^42^. In contrast, exposing urchins to nominal 0.1 μM copper combined with OA caused a slight but significant alkalosis of their coelomic fluid. This corresponded to slightly lower coelomic fluid *p*CO_2_ in the two copper treatments (alone and combined with OA) compared to their corresponding treatments without added copper (control and OA).

**Figure 2:**
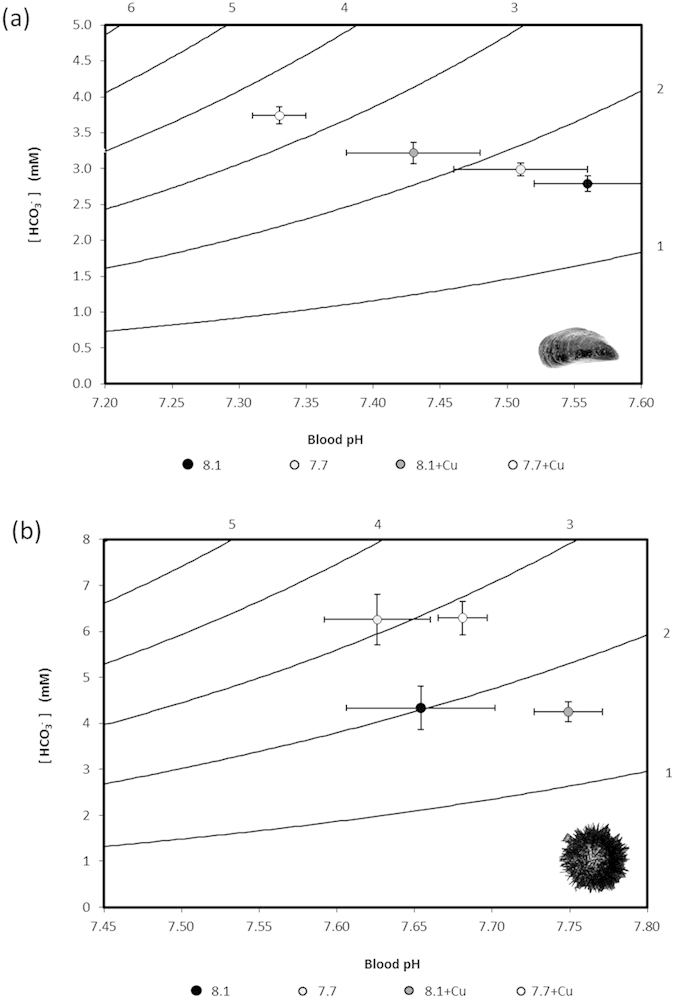
Davenport diagram illustrating the relationship between pH, bicarbonate and pCO_2_ in the haemolymph and coelomic fluid of (**a**) *Mytilus edulis* and (**b**) *Paracentrotus lividus* respectively. Lines represent isopleths of equal pCO_2_ (mmHg). Position calculated from means ± SEM for haemolymph/coelomic fluid pH and [HCO_3_^−^] according to pK_1_ values calculated from^57^.

These differences in acid-base responses between mussels and urchins to OA and copper exposures can be illustrated using Davenport diagrams (Fig. 2a,b). In mussels the decrease in haemolymph pH due to OA exposure is increased by the additional presence of copper with very little compensation from the elevation of bicarbonate ions. The pattern observed reflects a primarily respiratory acidosis of varying severity caused by the treatments. In urchins under OA conditions (with or without copper) the coelomic fluid pH is buffered by additional bicarbonate (i.e. a fully compensated respiratory acidosis) whilst copper caused a slight mixed respiratory/metabolic alkalosis. Our contrasting findings for mussels and urchins, and the contrasting findings of other studies across a number of aquatic species to similar copper concentrations^43^,^44^,^45^, suggest highly species-specific and concentration-dependent acid-base responses to copper.

### Oxidative stress responses also differed between mussels and urchins

**Figure 3:**
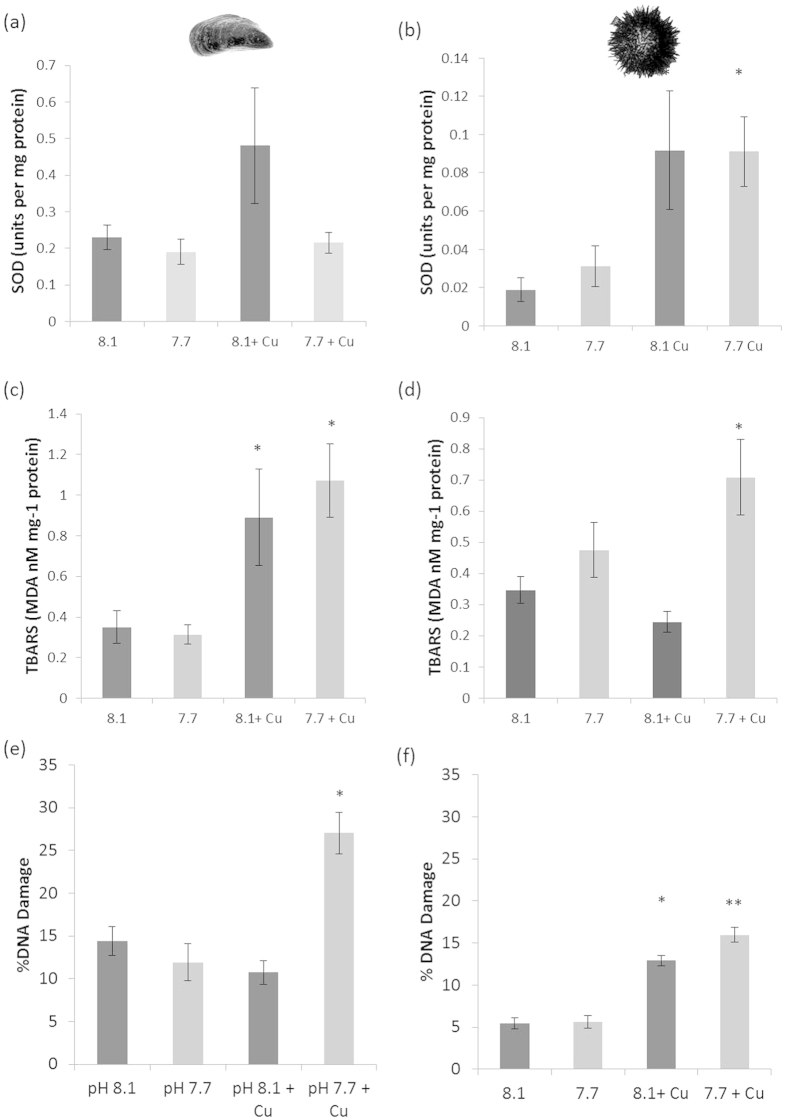
Oxidative stress indicators in the mussel *Mytilus edulis* (**a,c,e**) and the adult purple urchin *Paracentrotus lividus* (**b,d,f**) following 14 day exposures to elevated pCO_2_ with and without the presence of nominal 0.1 μM copper; (**a,b**) Activity of the anti-oxidant enzyme superoxide dismutase (SOD) activity; (**c**,**d**) lipid peroxidation measured as malondialdehyde (MDA) levels; (**e**,**f**) DNA damage, measured as percentage of single strand breaks in haemocytes/coelomocytes.

Superoxide dismutase (SOD) is an important cytosolic anti-oxidant enzyme. SOD activity has been shown to be induced in a number of marine invertebrate species in response to a range of environmental metals including copper^46^,^47^, with inhibition being reported for exposures using higher concentrations^48^,^49^. After 14 days exposure to our four treatments, SOD activity in mussel haemolymph showed no significant changes in response to any of these treatments, (Fig. 3a, two-way GLM model for copper: F_1,39_ = 2.57, P = 0.118; for OA: F_1,39_ = 3.15, P = 0.084; interaction term F_1,39_ = 1.73, P = 0.198). There was an average ~2 fold increase in SOD activity in response to copper alone compared to the treatments with no copper, however this response varied between individuals such that there was no significant difference overall. In urchins, however, SOD activity was significantly increased following exposure to nominal 0.1 μM copper under both ambient and OA conditions (Fig. 3b; two-way GLM model for copper F_1,32_ = 7.07, P = 0.013). OA conditions had no effect on extracellular fluid SOD activity in urchins (Fig. 3a; two-way GLM model for OA F_1,32_ = 0.05, P = 0.484; interaction term F_1,32_ = 0.08, P = 0.777).

Lipid peroxidation was significantly induced by exposure to copper in mussels under both ambient and OA conditions (Fig. 3c, two-way GLM model, for copper: F_1,39_ = 17.77, P < 0.001). There was no additional increase in lipid peroxidation when OA and copper were combined (two-way GLM model, for OA: F_1,39_ = 0.22, P = 0.640; interaction term F_1,39_ = 0.05, P = 0.486). Urchins again showed a different response, with no increase in lipid peroxidation induced by exposure to nominal 0.1 μM copper alone, most likely due to the protective effect of the increased SOD activity. A significant increase in lipid peroxidation was, however, induced by copper when exposed under OA conditions, despite the higher SOD levels (Fig. 3d; two-way GLM model for copper F_1,32_ = 0.54, P = 0.467; for OA F_1,32_ = 11.19, P = 0.002). No significant interaction term was present (interaction OA × copper F_1,32_ = 3.59, P = 0.068). This suggests a much greater toxicity effect of copper on lipids under the combined treatments that overwhelms the anti-oxidant defence capabilities in urchins in the combined OA and copper treatment.

Protein levels, measured as part of both the SOD and TBARS assays using the Bradford assay, were found to be ~20% lower in haemolymph/ coelomic fluid in response to copper exposure in both mussels and urchins independent of OA treatment. Mussels in the ambient pH and OA treatments were found to have haemolymph protein levels of 3.17 and 3.44 mg protein ml^−1^. Protein levels were significantly lower at 2.41 and 2.73 mg protein ml^−1^, respectively, in the treatments with the addition of nominal 0.1 μM copper (two-way GLM model for copper F_1,30_ = 8.26, P = 0.007). In urchins protein levels were generally slightly lower with 1.84 and 1.97 mg protein ml^−1^ in the ambient pH and OA treatments. The additional presence of copper lowered these levels to 1.46 and 1.56 mg protein ml^−1^, respectively, but this was not a significant effect (two-way GLM model for copper: F_1,30_ = 3.45, P = 0.073).

### DNA Damage was increased under OA in both species

Elevated copper induces DNA damage in the form of single strand breaks by the production of reactive oxygen species via the Fenton reaction and by base modifications such as 8-OHdG (a major product of DNA oxidation)^28^. In mussels copper-induced DNA damage was only observed in the combined OA and copper exposures, with no increase in DNA damage caused by exposure to nominal 0.1 μM copper alone (Fig. 3e). Combined exposure of adult mussels to both OA and copper in combination, however, resulted in a 1.9-fold increase in DNA damage from 14% DNA damage in the ambient (pH 8.1, no copper) treatment to 27% in the combined exposures. Statistical analysis reveals a significant interaction term between OA conditions and the presence of copper on DNA fragmentation (Two-way GLM model, for OA F_1,39_ = 12.54, P < 0.001; for copper F_1,39_ = 8.25, P = 0.007; interaction term F_1,39_ = 23.17, P < 0.001).

This indicates that mussels are able to cope with this low concentration of copper under the ambient pH/*p*CO_2_ conditions, either via their antioxidant defences preventing damage occurring in the first place or via efficient DNA repair activities. This does not hold true under OA conditions. The strong increase in DNA damage when exposed to nominal 0.1 μM copper under OA conditions has two likely explanations; a) the lack of anti-oxidant response (SOD) to the copper under OA conditions, and/or b) the increased availability and presumably uptake of the toxic free Cu^2+^ ion under the reduced seawater pH of OA conditions.

In urchins both OA and copper exposure were found to significantly affect DNA damage in coelomocytes. Exposure to copper induced a significant increase in DNA damage in both the pH 8.1 and pH 7.7 treatments, but as with mussels, DNA damage was significantly higher again in the OA treatment (Fig. 3f two-way GLM model for copper F_1,32_ = 138.12, P < 0.001; for OA F_1,32_ = 23.81, P = 0.038). No significant interaction term between copper and OA was found (F_1,32_ = 4.71, P = 0.104). This suggests that whilst increased SOD levels appear to provide some protection against lipid peroxidation it has not prevented copper-induced DNA damage from occurring in the urchin coelomocytes. This toxicity effect of copper on DNA is then significantly greater under OA conditions.

The urchins’ acid-base regulation strategy of increasing extracellular bicarbonate levels may actually act ‘protectively’ against copper toxicity and explain the overall reduced toxicity driven by the difference in the DNA damage response to combined copper and OA that we observed between mussels and urchins. It has been widely shown that copper is less toxic to freshwater species in hard water (i.e. higher alkalinity) than in soft water^50^. A protective effect of hypercapnia on copper toxicity has also been suggested by the work of Larsen *et al.*^51^ in the cod *Gadus morhua*. Free copper (II) ions (Cu^2+^) will form a range of complexes with bicarbonate ions such that the amount of free Cu^2+^ is only a small fraction of the total copper present in a high bicarbonate solution, with the relative concentrations of copper species varying with pH^52^. Elevated bicarbonate levels should therefore reduce the proportion of the toxic free copper ions and so reduce the amount that is bioavailable to cause damage. Whilst the damage to urchin’s lipids did not follow the same pattern as for DNA, this might be explained by their different positions within the cell, with the TBARS assay measuring mostly damage to lipids on the outside of cell membranes whilst DNA is within the cell nucleus, perhaps making lipids more susceptible to damage by the remaining free Cu^2+^ present. More detailed biochemical studies of the precise mechanisms of this damage would be required to fully explain these differences. A hypothesis resulting from this work requiring further testing could be that species which regulate their extracellular pH in response to OA by the elevation of internal bicarbonate levels will show reduced overall copper toxicity responses compared to those which are unable to acid-base regulate using bicarbonate.

This increased copper toxicity as a result of OA conditions in both test species is consistent with two other studies looking at metal-induced DNA damage under near-future OA. Roberts *et al.*^53^ found that DNA damage in the sediment dwelling amphipod *Corophium volutator* exposed to naturally contaminated sediments, which contained a range of metals and polycyclic aromatic hydrocarbons (PAHs), was higher under OA conditions than extant *p*CO_2_ conditions^53^. More recently an increase in copper-induced DNA damage in the sperm of the polychaete *Arenicola marina* was reported when worms were exposed to copper under elevated *p*CO_2_^54^. Synergistic toxicities have been reported between OA and copper in adults of the copepod *Amphiascoides atopus*^22^, where the LC_50_ for copper was reduced from 0.65 mg l^−1^ at pH 8.1 to 0.32 mg l^−1^ at a CO_2_-driven reduction of pH of 6.5. Increased copper toxicity under OA has also been reported for larvae of the polychaete worms *Pomatoceros lamarckii*^55^ and *Arenicola marina*^54^. Larvae are often considered to be the most sensitive life history stages to environmental stressors, particularly in free spawning marine invertebrates with bi-phasic life histories. We have now demonstrated that this increased toxicity under experimental OA can also be found in adult marine invertebrates at environmentally relevant concentrations.

## Conclusions

Our data has clearly shown that near-future OA significantly increases the sub-lethal toxicity responses of two key coastal marine invertebrates to coastally relevant concentrations of the common metal pollutant copper. We found that for both mussels (*Mytilus edulis*) and urchins (*Paracentrotus lividus*) copper-induced damage to DNA was significantly greater when animals were exposed to nominal 0.1 μM copper under OA (high *p*CO_2_/ low pH) conditions compared with animals exposed under extant *p*CO_2_ levels. This relative increase in copper-induced DNA damage under OA was four-times greater in mussels than in urchins (despite the measured seawater copper concentrations actually being lower in the mussel exposures than for the urchins). In mussels OA seemed to suppress the response of the anti-oxidant enzyme SOD to copper exposures, whilst in urchins lipid peroxidation was also increased when exposed to copper under OA. So whilst copper-induced toxicity was clearly increased in both species under near-future OA, there were slight differences in the biochemical details of how the two species responded.

Demonstrating an increase in the toxicity of copper in two ecologically and economically important coastal invertebrates under near future OA is a significant cause for concern given the widespread nature of coastal metal contamination. This is particularly the case given the relatively low concentrations of this coastal contaminant used in the present study, which are relevant to measurements of copper contamination for UK coastal waters generally and which are often exceeded in contaminated locations globally^20^,^21^. Furthermore, the changes in seawater copper speciation expected with a reduction in seawater pH will be compounded by an increase in the total copper released from sediments under OA conditions^15^. Subsequently the OA-induced increase in bioavailable copper is expected to be two-fold. We cannot fully determine whether these interactions between OA and copper are additive (the sum of the individual stressor effects) or synergistic interactions (greater than the sum of the individual stressor effects) due to the limited number of copper concentrations or seawater pH levels used here. However, the strong interaction terms in the analysis for some of the end points measured here are suggestive that synergies are likely to be present. Recent meta-analysis studies have shown that synergistic interactions between multiple abiotic stressors in the marine environment are more common than additive interactions^11^,^14^. This highlights a real need for the potential for interactions between climate driven stressors and coastal pollution to be studied in much greater detail rather than relying on predictive modelling approaches for environmental risk assessments.

Our results have clear implications for ecotoxicological assessments, highlighting a need to better understand how OA will alter the behaviour and toxicity of not just copper or metals generally but a wider range of environmental contaminants. Such knowledge is vital for those charged with protecting our marine habitats in order to provide justifiable predictions of OA impacts in coastal regions. In demonstrating a contrasting toxicity response in two ecologically important marine invertebrate species, related to acid-base regulation, we show that organismal responses may be far more diverse than either single stressor or single species studies have previously indicated. Furthermore, our results imply that not only may we be under-estimating OA impacts for coastal invertebrates exposed to chronic metal pollution, but also that OA may impact a much wider range of species, not traditionally considered as OA sensitive, due to this indirect effect on metal toxicity. This suggests that further investigations on OA-pollutant interactions in a wider range of organisms will be important to better understand the near future impact of toxic coastal contaminants for marine organisms globally.

## Methods

Adult *Paracentrotus lividus* specimens (4–6 cm diameter) were purchased from Dunmannus Sea Farm Ltd. in Cork, Ireland. Adult *Mytilus edulis* specimens were collected by hand from the intertidal range of the River Exe estuary, Exmouth, Devon, UK. Individuals were left for 7 days in 30 litre holding tanks at 15 °C in ambient artificial seawater (pH_NBS_ 8.1, 470 μatm *p*CO_2,_ salinity = 35) to acclimatise prior to the exposures. Ten individuals per treatment were exposed to one of the following four treatments for 14 days at 15 °C; (1) ambient conditions (pH_NBS_ 8.1) with no added copper; (2) ambient conditions (pH_NBS_ 8.1) with nominal 0.1 μM copper sulphate added; (3) OA conditions (pH_NBS_ 7.7) with no added copper; (4) OA conditions (pH_NBS_ 7.7) with nominal 0.1 μM copper sulphate added.

Seawater pH_NBS_ values of 7.7 were targeted to represent near-future OA treatments as projected according to scenario RCP 8.5 and the 2013 IPCC WGI AR5^4^,^56^; full seawater chemistry is provided in Tables 1 and 2. Seawater pH in the OA conditions was nominally maintained at pH_NBS_ 7.7 (to a resolution of 0.05 units) using pH computers (Aqua Medic, Bissendorf, Germany) which continually controlled the release of CO_2_ gas directly into the header tanks to maintain stable conditions throughout the experimental exposures. Partial water changes (50%) were carried out every 48 hours using temperature equilibrated water of the correct pH and CO_2_ level and copper concentrations were re-dosed appropriately. Seawater pH_NBS_ (Metrohlm 827 pH lab), temperature, and salinity (Mettler Toledo SG7), were measured daily in header tanks and experimental chambers. Water samples were taken every third day for measurements of dissolved inorganic carbon (DIC) and metals. Seawater DIC analysis was carried out as described in detail in Lewis *et al.*^55^ using a custom built system and using certified reference materials from Andrew Dickson’s Laboratory (Scripps Institution of Oceanography batch 114). Copper concentrations in seawater were determined using ICP-MS.

Following the 14 day exposure samples of extracellular fluid (haemolymph for mussels and coelomic fluid for urchins) were collected from each individual. Extracellular fluid for TCO_2_ analysis was stored in 100 μl hematocrit (micro capillary) tubes sealed with paraffin oil and the Critoseal^TM^ capillary tube sealant (Fisher) then analysed using a Corning 965 CO_2_ analyser (Corning Ltd., UK). Extracellular fluid for oxidative stress assays was snap frozen in liquid nitrogen and stored at −80 °C until analysis. Immediately following extraction extracellular fluid pH was measured at 15 °C using a pH meter (Hannah Instruments HI 8314) and microprobe (Cole Parmer, Accumet) calibrated prior to usage using pH_NBS_ 7.00 and 10.00 specific buffers (calibrated to 7.04 and 10.11 at 15 °C). Acid-base parameters were then calculated using a modified version of the Henderson-Hasselbalch equation using the first dissociation constant (pK) for carbonic acid and solubility constant (αCO_2_) for carbon dioxide derived from Truchot^57^.

Superoxide dismutase (SOD) activity was measured using the nitroblue tetrazolium (NBT) microplate assay^58^. Lipid peroxidation was determined using the thiobarbituric acid reactive substances (TBARS) assay^59^ which quantifies malondialdehyde (MDA), a secondary product of lipid peroxidation, via its reaction with thiobarbituric acid. DNA damage in haemocytes/ coelomocytes was measured as single strand DNA breaks using the comet assay. Two hundred microlitres of haemolymph/ coelomic fluid from each individual was used immediately after sampling for the comet assay according to the methods described by Lewis & Galloway^60^, using alkaline conditions at 5 °C.

Data were analysed using 2-way analysis of variance (ANOVA) general linear models with the fixed factors; ‘pH’ and ‘copper concentration’.

## Additional Information

**How to cite this article**: Lewis, C. *et al.* Ocean acidification increases copper toxicity differentially in two key marine invertebrates with distinct acid-base responses. *Sci. Rep.* **6**, 21554; doi: 10.1038/srep21554 (2016).

## Figures and Tables

**Table 1:** Seawater carbonate chemistry and copper levels for the four experimental treatment solutions for the *Mytilus edulis* exposures.

Treatment	Temperature (°C)	pH_NBS_	Salinity	Copper (μM)	TA (μmol/kg)^†^	TCO_2_ (μmol/kg)	pCO_2_(μatm)^†^	HCO_3_^−^ (μmol/kg)^†^	CO_3_^2−^ (μmol/kg)^†^	ΩCa^†^	ΩAr^†^
8.1	15.0 (±0.1)	8.14 (±0.01)	35.68 (±0.04)	0.016 (±0.006)	2345.8 (±68.6)	2132.4 (±60.4)	436.4 (±10.9)	1960.2 (±53.3)	156.0 (±7.7)	3.7 (±0.2)	2.4 (±0.1)
7.7	15.0 (±0.1)	7.68 (±0.01)	35.96 (±0.06)	0.011 (±0.002)	2303.1 (±63.6)	2264.3 (±63.3)	1373.0 (±69.2)	2152.5 (±60.2)	60.6 (±2.7)	1.4 (±0.1)	0.9 (±0.4)
8.1 + Cu	14.6 (±0.1)	8.09 (±0.01)	35.60 (±0.04)	0.047 (±0.007)	2635.3 (±55.0)	2423.1 (±38.4)	536.2 (±7.6)	2238.5 (±39.7)	164.1 (±7.1)	3.9 (±0.2)	2.5 (±0.1)
7.7 + Cu	14.5 (±0.1)	7.68 (±0.01)	35.74 (±0.07)	0.052 (±0.007)	2662.8 (±56.4)	2568.7 (±41.7)	1481.4 (±16.1)	2486.6 (±49.7)	73.0 (±3.1)	1.7 (±0.1)	1.1 (±0.1)

**Table 2:** Seawater carbonate chemistry and copper levels for the four experimental treatment solutions for the *Paracentrotus lividus* exposure.

Treatment	Temperature (°C)	pH_NBS_	Salinity	Copper (μM)	TA (μmol/kg)^†^	TCO_2_ (μmol/kg)	pCO_2_(μatm)^†^	HCO_3_^−^ (μmol/kg)^†^	CO_3_^2−^ (μmol/kg)^†^	ΩCa^†^	ΩAr^†^
8.1	15.6 (±0.1)	8.14 (±0.01)	34.06 (±0.02)	0.031 (±0.006)	2599.7 (±35.2)	2364.9 (±25.5)	470.7 (±15.8)	2168.9 (±19.2)	178.7 (±8.0)	4.3 (±0.2)	2.8 (±0.1)
7.7	15.6 (±0.1)	7.72 (±0.01)	34.11 (±0.02)	0.028 (±0.004)	2538.5 (±36.4)	2467.9 (±28.6)	1297.5 (±85.0)	2337.0 (±28.3)	83.3 (±12.4)	2.0 (±0.3)	1.3 (±0.2)
8.1 + Cu	15.3 (±0.1)	8.13 (±0.01)	34.25 (±0.03)	0.084 (±0.002)	2673.0 (±30.6)	2452.6 (±21.4)	528.4 (±17.9)	2262.0 (±15.8)	170.9 (±7.1)	4.1 (±0.2)	2.6 (±0.1)
7.7 + Cu	15.23 (±0.1)	7.71 (±0.01)	34.50 (±0.05)	0.088 (±0.005)	2655.1 (±22.9)	2608.8 (±21.4)	1483.1 (±31.9)	2481.3 (±20.2)	72.2 (±1.8)	1.7 (±0.1)	1.1 (±0.1)
